# Clinical Presentation and Management of Malignant Psoas Syndrome: A Scoping Review of Case Reports and Case Series

**DOI:** 10.7759/cureus.41522

**Published:** 2023-07-07

**Authors:** Delwin Suraj, Angel Zhang, Taylor Appelbaum, Nahiyan Ahmed, Susana Shih, Joseph Gofman, Klea Kalenja, Juanito N Abrigo, Valeriya Shaporova, Arhum Mannan, Robin J Jacobs

**Affiliations:** 1 Medicine, Nova Southeastern University Dr. Kiran C. Patel College of Osteopathic Medicine, Fort Lauderdale, USA

**Keywords:** opoids, mps, case reports, cancer, chronic and acute pain management, malignant psoas syndrome

## Abstract

Malignant psoas syndrome (MPS) is a rare and underreported clinical syndrome that significantly impacts the quality of life of cancer patients through metastatic infiltration of the iliopsoas muscle. Patients suffering from MPS often present with painful hip flexion, loss of mobility, and immense pain in their legs and back. The current literature describing the clinical presentation, management, and prognosis of MPS is limited primarily to case reports and outdated literature reviews. There remains a gap in the current knowledge of MPS and in the management of this complex cancer-related pain syndrome. Thus, this scoping review aimed to map current case reports and case series on MPS for clinical presentation, treatment modalities, and resulting prognoses of MPS in late-stage cancer patients. A systemized search using the databases Embase and PubMed (Medline) was conducted to access case reports and case series published between January 1990 and October 2022 that met the study’s inclusion criteria: (1) adult patients with metastatic cancer; (2) MPS symptoms secondary to infiltration of iliopsoas; (3) clinical presentation, treatment modality, and prognosis; and (4) English-language text. Our search strategy yielded 1926 citations. After removing 629 duplicates, 1,283 reports were excluded due to failure to meet eligibility criteria (n=1,271) or inaccessibility (n=12). Using the JBI appraisal tools for case reports and case series, a total of 14 articles remained for the final review. With histories of either genitourinary, hepatic, gastric, or skin cancer, each case reported new onset intense pain in the legs, back, abdomen, or pelvis with associated symptoms such as flexion of the hip or gait disorder. A computerized tomography (CT) scan, magnetic resonance imaging (MRI), or positron emission tomography (PET) scan typically confirmed metastasis into the iliopsoas causing these symptoms, which suggested MPS. Each case utilized two to seven different pain management strategies to alleviate MPS symptoms. Many cases first used opioids for pain relief. Following a necessitated increase in morphine equivalent daily dose, a subsequent increase in the strength of analgesic, change in route of administration, and integration of combination drug therapy were generally added to the treatment regime. Many cases reported successful management of symptoms through utilizing methadone, radiation therapy, botulinum toxin injection, increased opioid dosage, or epidural catheter administration of opioids. A unified clinical definition of MPS may be required to inform physicians of this syndrome to help support clinical decisions regarding treatments for patients. The studies indicated that a clearer guideline for treatment protocol may be warranted as most cases reported utilizing various treatment medication dosages and procedures with vastly differing results.

## Introduction and background

Malignant psoas syndrome (MPS) is a rare clinical syndrome that significantly impacts the quality of life of terminally ill patients through metastatic infiltration of the psoas major muscle secondary to advanced-stage malignancy [1-3]. First described by Stevens and Gonet in 1990, MPS was identified among four advanced-stage cancer patients between 1985 and 1989, each of whom shared a clinical presentation of painful fixed flexion of the ipsilateral hip, symptoms indicating proximal lumbosacral plexopathy, and CT or pathological evidence of malignant infiltration of the psoas major muscle [4]. Despite the severity of these symptoms, current literature describing the diagnostic criteria, management, and prognosis of MPS is limited primarily to case reports and a few outdated literature reviews [5]. There remains a gap in the current knowledge of MPS regarding the management of this complex cancer-related pain syndrome [2]. Thus, this scoping review will seek to map the clinical presentation, treatment modalities, and resulting prognoses of MPS as evidenced in the existing body of case reports.

The pathogenesis of MPS is associated with metastatic infiltration of the psoas major muscle and its surrounding structures. The psoas major contains five origins, including thoracic vertebrae 12 (T12) and lumbar vertebrae 1-4 (L1-4) [4,6]. The fusiform muscle travels inferolateral through the lacuna musculorum to insert on the lesser trochanter of the femur [4,7]. The innervation of the psoas major lies in the ventral rami of L1-L4 collectively termed the lumbar plexus [4,6]. Dynamically, the psoas major flexes the hip, allowing the thigh to travel along the plane of the pelvis [4,8,9]. Although an uncommon occurrence, malignant infiltration of this muscle and its surrounding plexus of nerves are a significant contributor to the classical symptoms associated with MPS [10,11]. These symptoms include proximal lumbosacral plexopathy, ipsilateral nociceptive pain, or painful ipsilateral hip flexion with a positive psoas muscle stretch test [1,2,4,5,12,13].

Healthcare professionals generally follow the World Health Organization (WHO) three-step analgesic ladder proposed in 1986 to manage this unique cancer-related pain syndrome [14]. This three-tiered approach builds on subsequent levels as the pain continues to persist or increases [14]. The base protocol includes non-opioids with or without the use of adjuvants [14]. The second-tier protocol adds weak opioids as a treatment option, and then the third-tier protocol adds the use of strong opioids [14]. Despite the strategy’s effective usage in managing cancer pain, MPS has been shown to be highly resistant to standard pain management protocol with practitioners relying on more aggressive treatment modalities such as chemotherapy, radiation therapy, nerve blocks, spinal analgesia, and surgery to alleviate their pain [1,5]. Despite treatment interventions, most patients die within one year of MPS symptom onset [5,15-18].

MPS is largely unreported with complex and varied treatments due to the limited case reports discussing clinical presentation, diagnosis, and management [5]. Thus, early recognition of MPS is imperative for predicting imminent mortality and initiating prompt pain control [19-21]. This can further guide the planning of end-of-life care and managing family expectations [20]. Following a timely diagnosis, appropriate and individualized treatment must be employed to manage this complex condition to provide more concise and direct relief for patients [18,22]. Thus, this scoping review will identify case reports of cancer patients with MPS and examine clinical presentation, treatment modalities, and the relative efficacy of management strategies.

## Review

Methods

Eligibility Criteria

To meet the goals of this scoping review, each article included a case report of patients over the age of 18 affected by MPS. Only case reports between January 1, 1990, and October 1, 2022, were included in this study because MPS was first recognized in 1990. Case reports were included if they showed cancer metastasis to the iliopsoas muscle, pain in the surrounding structures of the iliopsoas muscle, diagnostic criteria, and treatment modalities of MPS. All papers were required to be written in English to avoid mistranslations. Articles involving pediatric and animal patients were excluded due to a lack of screening and diagnostic criteria. Systematic reviews, economic evaluations, clinical guidelines, randomized control trials, case-control studies, cohort studies, and laboratory diagnoses were excluded to focus on compiling case reports alone. Gray literature was not considered as our scoping review only accounted for published case reports and case series.

Search Procedure

The search strategy involved search terms compiled after a preliminary search of relevant case reports from PubMed. Two scientific databases PubMed (Medline) and Embase (Ovid) were included. PubMed is published by the National Library of Medicine, while Embase is published by Elsevier; thus, they allowed us wide access to case reports. These databases include substantial scientific literature showcasing relevant oncological case reports of MPS. The search strategy was initially executed in PubMed (Medline) and then adapted to Embase. All searches were conducted on October 2, 2022.

Search Strategy

The population, concept, and context (PCC) framework was used to construct the research question, “What are the current clinical presentation, treatment modalities, and relative efficacy of management strategies for patients with metastatic cancer presenting with malignant psoas syndrome?” with P = adult patients with metastatic cancer, C = patients presenting with malignant psoas syndrome, and C = current diagnostic criteria, treatment modalities, and relative efficacy of management strategies. Two medical students independently developed and conducted the search using relevant keywords. Relevant keywords and synonyms included but were not limited to malignant psoas syndrome OR lumbosacral plexopathy AND cancer OR tumor AND treatment OR diagnosis were used to produce a well-defined search strategy (Table 1).

**Table 1:** Search strategy using PubMed (Medline) and adapted for Embase Limiters: The publication year 1990–Present Advanced Search

Iteration	Inclusion
S6	S1 AND S2 AND S3 AND S4 AND S5
S5	Psoas*[Title/Abstract] OR Iliopsoas*[Title/Abstract] OR lumbosacral*[Title/Abstract]
S4	Case*[Title/Abstract] OR present*[Title/Abstract] OR finding*[Title/Abstract] OR report*[Title/Abstract] OR diagnos*[Title/Abstract]
S3	Pain*[Title/Abstract] OR pallia*[Title/Abstract] OR therap*[Title/Abstract] OR analges*[Title/Abstract] OR treat*[Title/Abstract] OR symptom*[Title/Abstract] OR sign*[Title/Abstract] OR resect*[Title/Abstract]
S2	Cancer*[Title/Abstract] OR Malign*[Title/Abstract] OR tumor*[Title/Abstract] OR metast*[Title/Abstract] OR Adenocarcinoma[Title/Abstract] OR carcinoma[Title/Abstract] OR lymphom*[Title/Abstract]
S1	"Malignant Psoas Syndrome"[Title/Abstract] OR "skeletal muscle"[Title/Abstract] OR "Lumbosacral plexopathy"[Title/Abstract] OR Psoas*[Title/Abstract]

Given that MPS is a rare condition in the spectrum of cancer manifestations, there are limited case studies and case reports on the subject. As such, the publication year for included studies was broadened to 1990 to account for the limited articles that display this condition. Due to the rare presentation of the disease, case studies and case reports were favored for this scoping review.

Screening and Selection of Sources of Evidence

**Figure 1:**
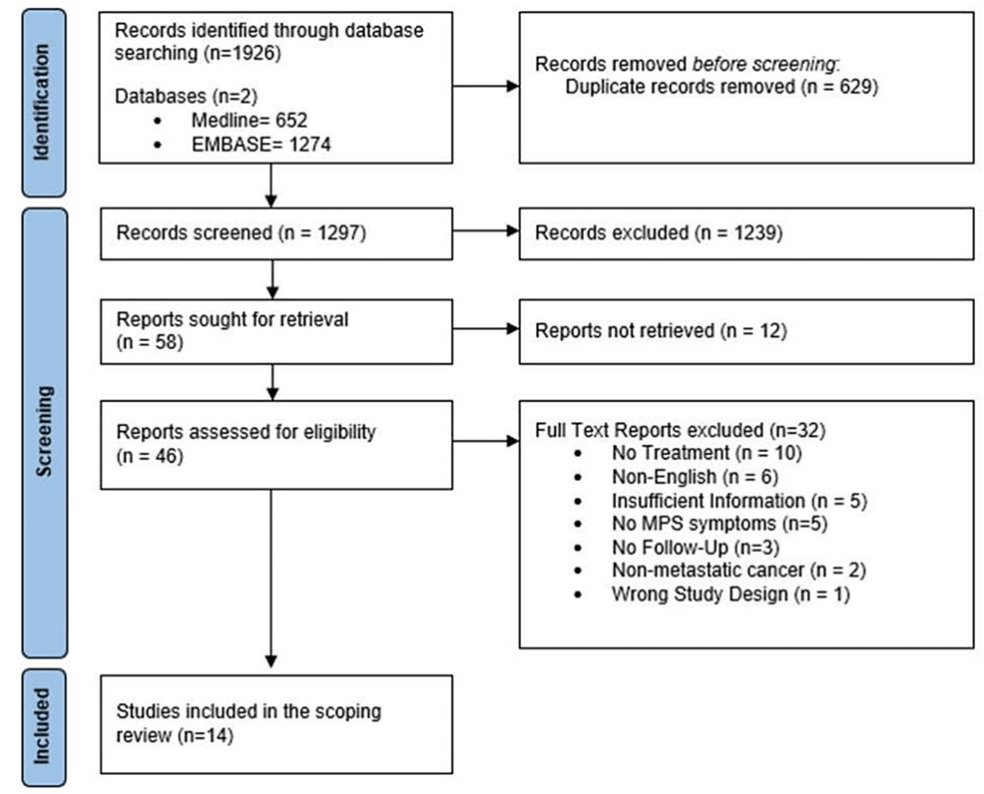
Figure 1: PRISMA Flow Diagram PRISMA: Preferred Reporting Items for Systematic Reviews and Meta-Analyses

The search strategy yielded a total of 1,926 citations, from which 629 duplicates were removed, resulting in a total of 1,297 studies for analysis. The research team reviewed each title and abstract and identified 58 articles relevant for additional appraisal. Among these, 12 articles were not accessible and were subsequently excluded. Two team members independently read and screened each of the 46 remaining articles in their entirety to confirm if they met the inclusion criteria. If there was disagreement in the screening choice between the two reviewers, a third team member was asked to review. Discussions continued between all three reviewers until a consensus was reached for each included article. Thirty-two articles were excluded for not meeting screening criteria: no treatment (n = 10), non-English (n = 6), insufficient information (n = 5), no MPS symptoms (n = 5), no follow-up (n = 3), non-metastatic cancer (n = 2), wrong study design (n = 1). The remaining 14 studies were included in the final review (Figure 1). 

Data Charting Process

Data regarding patient age, sex, primary cancer, cause of MPS, clinical presentation, treatment for MPS, treatment response, and survival time following MPS diagnosis were extracted from included case reports. Reviewers each received specific instruction on the quantitative and qualitative data needed to be obtained from each included study. This information was then inputted into a predesigned data charting form in Microsoft Excel (Microsoft Corporation, Redmond, Washington, United States). To confirm the accuracy of the data, four investigators then reviewed the inputted data for each of the 14 included articles. In the event of a dispute over data input, a discussion between the reviewers ensued until a consensus was reached. 

Results 

Overview of Clinical Presentation

In each of the 22 cases extracted from the 14 articles, pain in the leg, back, lower abdomen, or pelvis was reported ipsilateral to the infiltrated psoas or iliopsoas muscle [1,2,4,10,13,15-17,22-27]. Among these cases, three expressed paresthesia involving the upper leg and/or knee [10,23,24]. A gait disorder was exhibited in eight cases, with three involving pain with gait [1,4,15,17,22,27], Fixed flexion of the hip ipsilateral to the metastasis was reported in nine cases [1,13,15,17,22,25,27]. Seven cases showed limitation of movement, two of which involved pain and one involved weakness [1,15,22,24,25]. Four cases revealed other symptoms such as abdominal distension or leg edema [1,15,27]. Imaging in the form of a CT scan was used to identify lesions in 16 of the cases [1,2,4,10,13,15-17,22,24,26,27]. Imaging in the form of a chest X-ray was used in one case to identify lesions [16]. Imaging in the form of a PET scan was used to identify lesions in five of the cases [2,10,13,15]. Imaging in the form of an MRI scan was used to identify lesions in six cases [1,2,13,25,26].

Opioids

Opioids were extensively used in 19 cases and included the use of agents such as morphine, morphine sulfate, morphine hydrochloride, oxycodone, hydromorphone, fentanyl, methadone, and/or tramadol [1,2,4,15,16,22-26]. One case reported effective pain management and restoration of gait with morphine, oxycodone, and the concurrent use of anti-inflammatory agents and antidepressants [4]. With the addition of radiation therapy, this patient remained pain-free for the duration of her life [4].

Nine case reports demonstrated varied therapeutic outcomes following the use of opioid therapy in combination with alternate treatment modalities [1,15,22,23,26]. Following the ineffective usage of anti-inflammatory agents, anticonvulsants, and/or opioid combination therapy, three cases relieved pain and restored the range of motion by utilizing methadone in conjunction with radiation therapy, anti-inflammatory agents, chemotherapy, physical therapy, and/or oxycodone [15]. Three other cases achieved similar results of relieved pain and increased range of motion by utilizing epidural catheter administration of oxycodone with fentanyl, anesthetic, and/or oxycodone after attempting ineffective regimens of oxycodone, anti-inflammatory agents, and/or muscle relaxants [22]. After no response to opioid combination therapy, anti-inflammatory agents, anticonvulsants, and/or muscle relaxants, two cases experienced significant pain reduction with the use of radiation therapy supplemented with opioids, anesthetics, and anti-inflammatory agents [1,26]. Despite several unsuccessful rounds of opioid combination therapy, anti-inflammatory agents, surgical procedures, and anticonvulsants, one case managed to relieve their pain and anxiety with muscle relaxants, counseling, antidepressants, oxycodone, and hydromorphone [23].

Combination opioid therapy did not sufficiently reduce pain symptoms in nine of these cases despite concurrent usage of chemoradiation, anti-inflammatory medication, neuropathic pain agents, antipsychotics, and/or muscle relaxants [1,2,16,24,25,27]. Opioids used in these combination therapies included morphine, oxycodone, fentanyl, hydrocodone, hydromorphone, and/or methadone [1,2,16,24,25,27]. One case had mild pain control following the usage of continuous IV morphine infusion via an elastomeric pump [16].

Neuropathic Agents

Neuropathic agent drug classes such as serotonin-norepinephrine reuptake inhibitors (SNRIs), anticonvulsants, and antiepileptics were involved in the treatment of neuropathic pain of MPS in 12 cases [1,2,4,10,15,16,22-24,26]. Five cases utilized pregabalin as the main neuropathic agent [1,15,22]. Despite usage with opioids, anti-inflammatory agents, and other analgesics, pregabalin did not show a significant reduction in pain in the patients [1,15,22]. Three case reports utilized gabapentin as the main neuropathic agent [2,23,24]. One case showed sufficient improvement in pain management, but neuropathic symptoms of pain persisted [23]. It was unclear, however, if this improvement was a result of the neuropathic medication or the morphine that was concurrently administered [23]. Two cases showed no improvement in pain management with gabapentin despite the addition of morphine, muscle relaxants, or physical therapy [2,24].

Three cases utilized SNRIs with their treatment [2,23,24]. However, only one article showed that pain was well controlled. One article showed that venlafaxine had initially adequate pain control, but the pain returned after two weeks. One article utilized both gabapentin and duloxetine in the treatment of neuropathic pain [24]. Despite concurrent usage of opioids, this case did not experience a reduction in pain [24].

Four cases utilized epidural administration of local anesthetics with all displaying signs of pain resolution [22,26]. One case utilized lacosamide, an antiepileptic, but there was no information on its effectiveness [10]. Doxepin was used in one article and showed pain was effectively controlled and no pain with gain [4].

Cancer Treatment Drugs

Within the articles, eight case reports utilized chemotherapy in their treatment of MPS [1,2,10,15,17,17,23,25]. Following the ineffective use of opioids, non-steroidal anti-inflammatory drugs (NSAIDs), and analgesics, one case showed symptomatic improvement after the administration of chemotherapy agents paclitaxel, carboplatin, or a placebo/investigational drug [1]. The patient subsequently underwent radiation therapy and an additional round of salvage chemotherapy with paclitaxel, carboplatin, and bevacizumab, leading to tumor remission and total loss of symptoms [1]. Another case demonstrated partial improvement of psoas muscle involvement following a transition in chemotherapy treatment from bicalutamide to leuprolide and docetaxel in conjunction with an anticonvulsant and steroid [10]. Utilizing chemotherapy, radiation, and methadone, a third case reported well-controlled pain after unsuccessful rounds of opioids, anticonvulsants, and NSAIDs [15].

Despite concurrent usage of opioids, NSAIDs, antidepressants, anticonvulsants, and/or other neuropathic agents, three cases showed worsening symptoms of weakness, edema, decreased motion of the hip, and/or pain following the usage of chemotherapy [1,2,25]. One case demonstrated no improvement in pain management after combining chemotherapy with other treatment modalities such as analgesics, steroids, surgery, radiation, clinical trial participation, and nerve block [23]. One case had inconclusive results as findings showed an initial reduction of tumor size and then a prompt increase in tumor size, deep vein thrombosis (DVT), and bilateral hip contracture with additional cycles of chemotherapy [17].

Muscle Relaxants

Muscle relaxants such as benzodiazepines, baclofen, and botulinum toxin injections were used in five cases for MPS relief [2,22,25,26]. Two cases reported improvement in pain symptoms with the use of muscle relaxants [25]. After ineffective usage of opioids, chemoradiation, anti-inflammatory agents, anesthetic agents, and/or nerve root blocks, two cases reported long-term improvement in pain symptoms and movement after a botulinum toxin injection was administered to the iliopsoas muscle [25].

Despite synchronous usage of muscle relaxants with agents such as opioids, radiation therapy, chemotherapy, anti-inflammatory agents, SNRIs, anticonvulsants, and anesthetic agents, three cases reported no improvement in pain symptoms with the usage of muscle relaxants including flunitrazepam, diazepam, and clonazepam [2,22,26].

Anti-inflammatory Agents

Fourteen cases included the use of anti-inflammatory agents such as glucocorticoids and NSAIDs in the treatment of MPS [1,2,4,10,15,22,23,25,27]. Among these cases, two showed improvement in muscle involvement and/or pain relief with the concurrent use of chemotherapy, anticonvulsants, and hormone therapy for one case and the addition of opioids and antidepressants for the other [4,10].

Two cases reported mixed results of no improvement, worsening of pain, and/or alleviation of symptoms [1,15]. Of these, one case showed no improvement or worsening of symptoms with anti-inflammatory agents when used with opioids and analgesics, but symptoms were resolved with the additional usage of radiation therapy and analgesics [1]. The second case showed initial persistence of pain with anti-inflammatory use with the concurrent use of opioids but concluded with a reduction of pain levels after the addition of more opioid treatment [15].

One case reported worsening symptoms of pain following the usage of anti-inflammatory agents despite concurrent usage of chemotherapy, analgesics, opioids, antidepressants, and muscle relaxants [2].

Nine cases reported no changes in pain level after administration of anti-inflammatory agents despite concurrent medications such as opioids, chemotherapy, anticonvulsants, analgesics, and/or antipsychotics [1,15,22,23,25,27].

Surgical Procedures and Nerve Blocks

The surgical treatment of MPS was documented in three cases [13,23,24]. This modality of therapy offered mixed efficacy for interventional results in the management of MPS. One case using computed tomography-guided cordotomy to ablate the right spinothalamic tract provided only temporary relief, with the pain returning during bouts of activity [23]. Another case that completed a subtotal L4 vertebrectomy and resection of the left psoas muscle at L3-L5, resulted in a disease-free status for three years [13]. The patient still, however, faced post-operational complications such as ipsilateral weakness of the knee and hip adductor, as well as paresthesia of the thigh and medial lower leg [13]. One case that implanted a spinal cord stimulator patch showed dramatic pain relief [24].

Four cases utilized nerve blocks in their management [23-25]. One case used a hypogastric nerve block alongside a percutaneous cordotomy but still showed persistence of pain [23]. Two cases utilized lumbar nerve blocks; however, both were not effective in long-term pain relief with one case having persistent pain and one having pain relief for 24 hours only [24,25]. Two cases completed psoas compartment blocks with ropivacaine that helped alleviate pain only for one week [25].

Radiation Therapy

Radiation therapy was documented in 13 cases [1,4,10,13,15,22,23,25-27]. Eight cases reported improvement in pain relief, the recovery of normal gait, and/or tumor reduction after radiation therapy [1,4,13,15,26,27]. In two of these cases, radiation therapy was used alongside other medications including NSAIDs, an investigational drug or placebo, chemotherapy, opioids, and/or an analgesic with patients experiencing well-controlled pain symptoms or improvement [15,26]. The pain was reduced with sole radiation therapy in one case [13]. Five cases showed improvements with sole radiation therapy after having tried the use of NSAIDs, opioids, steroids, chemotherapy, antipsychotics, anticonvulsants, and/or antidepressants [1,4,27].

Four cases reported no improvement with persisting pain despite utilizing radiation therapy in conjunction with opioids, anti-inflammatory agents, nerve blocks, and muscle relaxants [22,23,25]. The outcome of one case that utilized radiation therapy was inconclusive as no treatment response was provided after the usage of this treatment modality [10]. The characteristics of the articles in the review are summarized in Table 2. 

**Table 2:** Summary table of the case reports and case series included in the review F: female; M: male; CT: computer tomography; DVT: deep vein thrombosis; MRI: magnetic resonance imaging; PET: positron emission tomography; LN: lymph node; NSAID: non-steroidal anti-inflammatory drug; IV: intravenous; T: thoracic vertebrae; L: lumbar vertebrae; SCS: spinal cord stimulator; NRS: numeric rating scale

Reference	Age and Sex of Participant	Primary Cancer	Clinical Presentation	Imaging	Cause of MPS	Treatments for MPS	Treatment Response	Survival Time Following MPS Diagnosis	
De La Cruz et al., 2013 [23]	48 F	Ovarian cancer	Left flank pain Left thigh pain and paresthesia	Not reported	Metastases to left psoas muscle via supraclavicular and retroperitoneal LN	(1) Morphine, methadone, hydromorphone, hypogastric nerve block, epidural steroid injection, percutaneous cordotomy	(1) Persistence of pain	Unknown (in hospice)	
						(2) Hydromorphone	(2) No treatment response information provided		
						(3) Surgical treatment, radiation therapy, chemotherapy, clinical trial participation	(3) Persistence of pain		
						(4) Morphine, physical therapy, gabapentin	(4) Pain sufficiently treated, still had nociceptive/ neuropathic pain with increasing numbness of the anterior thigh and sharp episodic pain		
						(5) Methadone, hydromorphone, hypogastric nerve block, radiation therapy, cordotomy	(5) Persistence of pain, opioid-induced neurotoxicity, light decrease in pain at rest with an escalation of pain with activity		
						(6) Oxycodone, hydromorphone, escitalopram, olanzapine, counseling	(6) Pain well controlled, anxiety managed		
Kalangara and Singh, 2018 [24]	68 M	Melanoma	Left leg pain and numbness Weak left leg flexion and knee extension Left leg edema Erythema and inflammation left groin	CT	Metastases to left psoas muscle via supraclavicular and retroperitoneal LN	(1) Hydrocodone, oxycodone, gabapentin, duloxetine	(1) Persistence of pain due to side effects	11 months (after SCS placement)	
						(2) Acetaminophen, fentanyl transdermal patch, hydromorphone, topical lidocaine 5% patches	(2) Persistence of pain		
						(3) Lumbar sympathetic block targeted at L3, fentanyl patch	(3) Paid reduced by 50% for 24 hours		
						(4) SCS trial targeted at T10 for 5 days, fentanyl patch	(4) 75% pain relief with return to normal activities, adequate paresthesia to painful areas in the left groin and anterior thigh		
						(5) Permanent SCS implant at T10, fentanyl patch	(5) 85% pain relief, improved function, and increased activities; pain fairly controlled at 8 months with SCS		
Kim and Choi, 2022 [25]	39 F	Cervical cancer	Left pelvic pain Left thigh pain Fixed flexion of left hip	MRI	Metastasis to left psoas muscle fascia via abdominal LNs	(1) Morphine sulfate, chemoradiation, oxycodone, NSAIDs	(1) Persistence of pain, unable to extend left hip or sleep	Unknown	
						(2) Epidural steroid injection at L2/L3 level	(2) Persistence of pain		
						(3) Psoas compartment block with ropivacaine	(3) Pain decreased from 8-9 NRS to 3-4 NRS, pain returned after one week aggravated by extension of the hip		
						(4) Botulinum toxin injection to psoas muscle	(4) Pain decreased to NRS 3 in two weeks, able to perform full hip extension, sleep, and walk comfortably, pain relief continued for nine weeks		
Kim and Choi, 2022 [25]	68 M	Hepatic cell carcinoma	Right inguinal thigh pain Right buttock pain Painful limitation of motion	MRI	Metastasis to right psoas and iliacus muscles	(1) L4 nerve root block, palliative radiotherapy to iliac bone, fentanyl patches	(1) Persistence of pain	Unknown	
						(2) Psoas compartment block with ropivacaine	(2) Pain decreased for one week, and upon returning was aggravated by walking or hip extension		
						(3) Botulinum Toxin Injection to iliacus and psoas muscle	(3) Improvement seen after 2 weeks, able to fully extend the right hip, walk independently, pain decreased to 3-4 NRS, relief persisted for 12 weeks		
Kong et al., 2021 [10]	61 M	Prostate cancer	Left paraspinal lumbar pain Left knee paresthesia Increased urinary frequency	CT PET	Metastases to left psoas muscle	(1) Bicalutamide, lacosamide, dexamethasone	(1) No treatment response information provided	Alive (at the time of original publication)	
						(2) Leuprolide, docetaxel, pegfilgrastim, lacosamide, dexamethasone	(2) PSA value improvement, partial improvement of psoas muscle involvement, additional enhancement of adjacent areas seen on imaging		
						(3) Radiation therapy to L3-S1 region	(3) No treatment response information provided		
McKay et al., 2017 13]	68 F	Sarcoma of uncertain classification	Low back pain radiating down to left leg Fixed flexion of left leg (+) reverse straight leg raise test	CT MRI PET	Metastasis to left psoas	(1) Radiotherapy to the abdominal and pelvic portion of psoas muscle and tumor mass	(1) Significant pain improvement with improved mobility and quality of life	Alive (3 years post-surgery)	
						(2) L4 vertebrectomy, left psoas muscle resected from L3 to L5 with resection of the left obturator nerve, genitofemoral nerve, L4 spinal nerve, and L4 root of femoral nerve	(2) Weakness of knee and ipsilateral hip adductor, patient remained disease-free for 3 years after surgery		
Mollica et al., 2019 [16]	60 F	Non-Small Cell Lung Cancer NSCLC	Lower back pain Left leg pain	CT Chest X-ray	Metastasis to left psoas muscle	(1) analgesics and opioids (morphine)	(1) Poor relief of pain due to recurrent breakthrough pain episodes	30 days (after admission)	
						(2) continuous IV morphine infusion via an elastomeric pump	(2) Mild pain control		
Ota et al., 2017 [26]	45 M	Gastric cancer	Back pain	CT MRI	Metastasis to left psoas muscle via abdominal paraaortic LNs	(1) Oxycodone, acetaminophen	(1) Persistence of pain	19 days (after completing radiotherapy)	
						(2) Oxycodone, flunitrazepam	(2) Persistence of pain		
						(3) Epidural catheter for levobupivacaine and lidocaine	(3) Pain decreased for over 10 minutes to obtain CT		
						(4) Oxycodone, acetaminophen, epidural levobupivacaine, oxycodone for breakthrough pain, radiotherapy. Bolus lidocaine or levobupivacaine before radiotherapy. Removal of the epidural catheter on day 15; completion of radiotherapy on day 16.	(4) Pain decreased to 3/10 (NRS) on day 16, worse with recumbency and supine position		
Stevens and Gonet, 1990 [4]	60 F	Bladder transitional cell carcinoma	Gait disorder Left leg pain radiating to groin Positive psoas test	CT	Metastasis to left psoas muscle via paraaortic LNs	(1) morphine, prednisolone, diclofenac, doxepin, oxycodone	(1) Pain effectively controlled, no pain with gait	5 months	
						(2) radiation therapy	(2) Pain-free with some recurrent lower extremity edema		
Stevens et al., 2010 [2]	53 F	Squamous cell carcinoma of the uterine cervix	Right groin allodynia and hyperalgesia Lower abdominal wall pain Right thigh pain	CT PET	Metastasis to right psoas muscle via pelvic and paraaortic LN	(1) Chemotherapy, morphine, acetaminophen, dexamethasone, venlafaxine, clonazepam	(1) Initially adequate pain control, over the next 2 weeks pain and distress rapidly increased	4 months (after MPS diagnosis)	
									(2) Methadone, hydromorphone rescue; gabapentin, midazolam, baclofen titration	(2) Minimum sustainable pain relief with titration	
						Takamatsu et al., 2018 [1]	31 F		Squamous cell cervical cancer	Gait disorder Left lower back pain radiating to hip, thigh, and knee Painful flexion of left hip	MRI	Metastasis to left psoas muscle via bilateral paraaortic LN	(1) tramadol, loxoprofen	(1) Persistence of pain	8 months (after symptom onset)	
(2) oxycodone, naproxen, acetaminophen, pregabalin	(2) Persistence of pain														
(3) oxycodone, naproxen, acetaminophen, pregabalin, betamethasone	(3) Persistence of pain														
(4) Intensity-modulated radiation therapy to psoas muscles	(4) Pain reduced gradually and recovered normal gait														
(5) Opioid unspecified regimen	(5) Pain well-controlled for 4 months after discharge														
Takamatsu et al., 2018 [1]	63 F	Uterine endometrial serous adenocarcinoma	Gait disorder Right leg weakness, edema, burning sensation, and pain Fixed flexion of right hip	CT	Metastasis to right psoas muscle via right paraaortic LN	(1) Oxycodone, loxoprofen, and acetaminophen	(1) Unspecified response	7 months (after symptom onset)	
						(2) Oxycodone, pregabalin, olanzapine, betamethasone	(2) Persistence of pain		
						(3) External beam radiotherapy to the lesion	(3) Pain improved, normal gait recovered, no MPS symptoms recurred		
Takamatsu et al., 2018 [1]	50 F	Fallopian tube cancer	Left lower back pain radiating to the lower leg Fixed flexion of left hip Abdominal distension	CT	Metastasis to left psoas muscle via bilateral paraaortic LN	(1) oxycodone, naproxen, acetaminophen	(1) Persistence of pain with intolerable side effects of increased opioid use	Alive (at time of original publication)	
						(2) fentanyl, naproxen, acetaminophen	(2) Worsening of pain		
						(3) paclitaxel and carboplatin chemotherapy, phase 3 clinical trial with either placebo or investigational drug	(3) Tumor remission achieved, symptoms disappeared		
						(4) Salvage chemotherapy with paclitaxel, carboplatin, bevacizumab	(4) Pain progressively subsided and patient had no disease symptoms		
Takase et al., 2015 [15]	59 M	Prostate cancer	Painful gait Back pain Left abdominal pain Right thigh unable to extend Positive psoas test on the right side	CT PET	Metastasis to right iliopsoas muscle via right common iliac LN	(1) Fentanyl, morphine hydrochloride hydrate, dexamethasone	(1) Persistence of pain	6 months (after methadone initiation)	
						(2) Etodolac, fentanyl, morphine hydrochloride hydrate, dexamethasone	(2) Persistence of pain		
						(3) Methadone	(3) Pain resolved, pt was able to extend thigh and walk		
Takase et al., 2015 [15]	35 M	Urachal cancer	Painful gait Left leg pain radiating to groin Left leg edema Fixed flexion of left thigh Positive psoas test on the left side	CT	Metastasis to left iliopsoas muscle via left common iliac LN	(1) Tramadol hydrochloride	(1) Persistence of pain	Alive (undergoing chemotherapy at the time of original publication)	
						(2) Oxycodone SR, pregabalin, loxoprofen sodium hydrate, oxycodone rescue	(2) Persistence of pain		
						(3) Methadone, oxycodone rescue	(3) Symptoms improved, decreased pain with walking and laying on the bed		
						(4) Radiation therapy, chemotherapy, and methadone	(4) Pain well-controlled		
Takase et al., 2015 [15]	70 F	Cervical cancer	Gait disorder Left lower abdominal pain radiating to the left groin and thigh Left thigh unable to extend Positive psoas test on left side	CT PET	Metastasis to left iliopsoas muscle	(1) oxycodone SR, loxoprofen	(1) Persistence of pain	2 months (after methadone treatment initiation)	
						(2) oxycodone	(2) Persistence of pain		
						(3) fentanyl	(3) Persistence of pain		
						(4) methadone, physical therapy	(4) Pain improved both at rest and with motion, patient able to extend the thigh		
						(5) methadone, loxoprofen	(5) Pain levels reduced		
Tsuchiyama et al., 2019 [17]	58 F	Bladder cancer	Gait disorder Right leg pain Fixed flexion of hip bilaterally	CT	Metastases to bilateral iliopsoas and pelvis muscles via extraperitoneal LN	(1) Gemcitabine, Carboplatin, Warfarin	(1) 10% reduction in tumor size	8 months (after symptom onset)	
						(2) Additional systemic chemotherapy cycles	(2) Increase in tumor size, increase in DVT, increased bilateral hip joint contracture		
Sanuki et al., 2022 [27]	49 F	Ovarian cancer	Fixed flexion of left hip	CT	Metastasis to bilateral iliopsoas muscle via surrounding enlarged LNs	(1) Hydromorphone	(1) Persistence of pain	Unknown	
						(2) Diclofenac	(2) Persistence of pain		
						(3) Loxoprofen sodium hydrate	(3) Persistence of pain		
						(4) Radiation therapy to the left side	(4) Able to lie supine, pain on left side decreased while right side increased		
						(5) Radiation therapy to right side	(5) Durable pain relief bilaterally		
Sanuki et al., 2022 [27]	54 F	Uterine Cancer	Gait disorder Right leg edema Fixed flexion of right hip Low back pain Right thigh pain	CT	Metastasis to right iliopsoas muscle	(1) Hydromorphone	(1) Persistence of pain, 4 NRS	Unknown	
						(2)Radiation therapy	(2) Pain immediately resolved and maintained for 17 months, 0 NRS		
Yamaguchi et al., 2017 [22]	68 F	Squamous cell carcinoma of the skin	Left thigh pain, exacerbated with extension Gait disorder (+) left psoas stretch test	CT	Metastasis to left iliopsoas muscle via left common iliac LN	(1) Oral oxycodone, loxoprofen, pregabalin	(1) Persistence of pain, disturbed walking	2 weeks (after discharge)	
						(2) Oral to epidural catheter L3/L4 opioid administration, fentanyl, ropivacaine	(2) Pain with rest resolved, able to extend left hip, able to ambulate with an aid		
Yamaguchi et al., 2017 [22]	61 F	Bladder cancer	Right back pain Right thigh pain (+) right psoas stretch test	CT	Metastasis to right psoas muscle via right retroperitoneal LN	(1) Acetaminophen, increased oral oxycodone dosage, diazepam, radiation therapy to retroperitoneal LN and right psoas muscle lesion	(1) Persistence of pain with extension of right hip and ambulating due to nausea and somnolence	3 months (after transfer to palliative care unit)	
						(2) Switch oral to intrathecal catheter L2/3 opioid admin, added morphine, bupivacaine	(2) Pain resolved, and normal gait recovered		
Yamaguchi et al., 2017 [22]	39 F	Cervical cancer	Pain with right hip extension Fixed flexion of right hip (+) right psoas stretch test Right thigh hypoesthesia	CT	Invasion of right psoas muscle by cervical cancer lesion in pelvis	(1) IV morphine switched to oral oxycodone, other analgesics (celecoxib, acetaminophen, betamethasone, pregabalin), and increased dosage	(1) Persistence of pain, patient unable to remain in the supine position	6 weeks (after epidural treatment initiation)	
						(2) Switched from oral to epidural catheter L3/L4 opioid administration, fentanyl, ropivacaine	(2) Pain improved, and the patient able to lie in the supine position		

Discussion 

Diagnostic Criteria of MPS

MPS was originally detailed by Stevens and Gonet in 1990 as a distinct cancer-associated condition that could be diagnosed with the presence of the following: (1) clinical evidence of proximal lumbosacral plexopathy, (2) painful fixed flexion of the ipsilateral hip with worsening pain with hip extension, and (3) CT or pathological evidence of ipsilateral psoas major muscle invasion [4].

The case reports highlighted in this review showcased patients with a history of metastatic cancer originating predominantly from the genitourinary tract. The symptomology reported from each article suggests that proximal lumbosacral plexopathy associated with MPS typically manifests as intractable pain in the leg, back, lower abdomen, or pelvis ipsilateral to the infiltrated iliopsoas muscle. In contrast with the original diagnostic criteria of MPS, our results showed that not every case with MPS presented with fixed flexion of the hip and that this criterion could be expanded to also include symptoms of gait disorder and limitation of movement. In addition, our results found that MPS symptoms could be attributed to malignant infiltration of the iliacus and psoas minor muscles, rather than solely psoas major muscle infiltration. The cases in this review also showed that infiltration of the iliopsoas muscle could be identified through CT, MRI, or PET, rather than solely CT.

The case reports within this review highlighted the importance of a prompt diagnosis for MPS. From 20 cases, we found that seven cases were not diagnosed with MPS during their treatment even though their patient’s symptoms aligned with those of the syndrome. These cases often had more delayed or ineffective management of MPS symptoms compared to other case reports that recognized the condition and initiated aggressive pain relief strategies sooner. This indicates that further education on the syndrome is needed so that clinicians can quickly identify this debilitating disease and improve the quality of life of their patients.

Management of MPS

The WHO recommended a simple three-step analgesic ladder for pain management in adult cancer patients [14]. Step one involved the use of non-opioids such as NSAIDs in conjunction with adjuvants like muscle relaxants, antidepressants, anticonvulsants, and steroids [14]. If pain persisted, clinicians could move on to step two, which involved the use of weak opioids like codeine or tramadol [14]. In the presence of severe, unrelenting pain, step three included the use of strong opioids such as morphine, oxycodone, or fentanyl [14]. The cases within this review generally follow this stepwise approach, often using two to seven different management strategies, in treating the intractable and debilitating pain associated with MPS.

Step 1: Non-Opioids and Adjuvants: Fourteen case reports employed anti-inflammatory agents, like NSAIDs and steroids, as part of their treatment for MPS. Four patients reported improvement in MPS symptoms at one point of their treatment with the use of anti-inflammatory agents in conjunction with either neuropathic agents, cancer treatment drugs, or opioids [1,4,10,15]. Nine cases, however, demonstrated no improvement or worsening of symptoms [1,2,22,23,25,27]. This suggests that anti-inflammatory agents may not be an effective means of management for MPS-associated pain. This observation is in contrast with several studies that have shown the efficacy of NSAIDs in reducing pain and lowering opioid usage in advanced cancer patients [28-30]. Our findings for the utilization of corticosteroids on MPS are similar to those of two reviews, which showed that corticosteroids may provide only a moderate analgesic effect on cancer pain [31,32].

Although 12 cases utilized neuropathic agents such as SNRIs, anticonvulsants, and antiepileptics, a majority of the patients in these cases did not experience a reduction in pain following the usage of these agents [1,15,22]. The three cases that did experience improvement with neuropathic agents, however, were all administered opioids such as methadone, hydrocodone, and morphine in conjunction with the neuropathic agent [2,23,24]. This is consistent with a meta-analysis on opioids and gabapentin combination therapy, which showed that the pain intensity of patients using both drugs was significantly less than that of sole opioid administration [33]. The combination of different treatment modalities in controlling pain in MPS patients is a common practice but may impose a challenge to discerning which treatment combination is responsible for providing relief. A study on the efficacy of amitriptyline, gabapentin, and pregabalin showed that while all three agents were successful in the treatment of neuropathic cancer pain, pregabalin was found to have the most significant efficacy in relief of symptoms [34]. This implies that research on pregabalin’s analgesic properties and its combination with opioids should be further explored due to its synergistic effects.

Five cases used muscle relaxants such as diazepam, baclofen, and botulinum toxin injections for the management of MPS [2,22,25,26]. The three cases that utilized diazepam reported no improvement in pain symptoms despite concurrent usage of either strong opioids, anti-inflammatory agents, radiation therapy, or neuropathic agents [2,22,26]. Two cases, however, experienced a substantial restoration of movement and reduction in pain with the use of botulinum toxin injection alone [25]. Our findings are consistent with several published studies that demonstrate the improvement in pain intensity after minimally invasive botulinum toxin injections [35-37]. Although the use of botulinum toxin is limited to two case reports, the considerable decrease in MPS symptoms may indicate the need for further research into its efficacy. Intractable pain is not a unique association with invasive metastatic cancers like MPS. The suspected mechanism of botulinum toxin pain relief is through inhibiting acetylcholine neurotransmitter release [35-37]. Inhibition at the neuromuscular junction results in pain relief from muscle spasms while relief of neuropathic pain is from inhibition of neurotransmitters both peripherally and centrally [38].

Steps 2 and 3: Opioids: Tramadol, a weaker opioid, was utilized in two cases and did not result in an alleviation of MPS symptoms [1,15]. Seventeen cases utilized strong opioids in conjunction with various other treatment modalities for MPS pain management [1,2,4,15,16,22-26]. Of these cases, 10 reported an improvement in symptoms at one point with morphine, methadone, hydromorphone, oxycodone, fentanyl, or epidural administration of opioids in conjunction with other therapeutic agents [1,2,15,22,23,26]. Among the opioids used, the reports in this review suggest that methadone and epidural administration of opioid lead to the most direct reduction in MPS symptoms with the least usage of concurrent medication.

Methadone was employed in the management of five cases. Among these cases, three showed a significant decrease in MPS symptoms following the use of methadone in conjunction with either physical therapy, radiation therapy, or an anti-inflammatory [15]. This aligns with several studies that extensively showed that methadone can be a viable option in managing unrelenting cancer pain while also lowering the need for additional use of opioids [39-42]. 

Epidural administration of opioids was implemented in the management of MPS symptoms in three cases. These three cases demonstrated significant pain relief using epidural administration of opioids in combination with anesthetics and oral opioids, such as fentanyl or morphine [22]. The epidural route of opioid administration has been effective in treating cancer-related neuropathic pain [43]. One study on interventional pain management for cancer-related pain revealed that pain-targeted neuraxial techniques such as epidural opioid therapy optimize pain relief due to the close proximity to the pain source [43]. This allows physicians to optimize pain relief while also minimizing side effects and the dosages required for therapy [43]. In another study discussing analgesic intervention, the reduced side effect profile of using epidural administration compared to using systemic oral opioids allows for decreased opioid treatment dosages in cancer pain management [44]. As revealed in our three cases using epidural opioids for persistent MPS pain, the pain cannot be treated with epidural treatment with a single opioid regimen [22,45-47]. Instead, it is recommended that combination therapy with opioids and local anesthetics has been effective in treating MPS pain as indicated in the presented cases [22,45-47].

Nine cases in our review, however, did not provide substantial pain relief using morphine, fentanyl, oxycodone, hydrocodone, hydromorphone, and methadone in combination with either chemotherapy, anti-inflammatory agents, surgical procedures, radiation therapy, neuropathic agents, and muscle relaxant [1,2,16,24,25,27].

Other Treatment Modalities

Thirteen cases showed that radiation therapy led to improved pain relief, normal gait recovery, and/or tumor reduction after radiation therapy [4,10,15,22,23,25-27]. Seven case studies showed that radiation therapy was effective in conjunction with NSAIDs in reducing pain symptoms [1,15,26]. Six cases showed that radiation therapy alone was effective in reducing pain symptoms often after aggressive pharmacological methods were shown to be ineffective [1,4,13,27]. These findings suggest that radiation therapy is effective in pain management as a part of palliative care. However, more research must be done to explore if radiation is effective in pain management on its own or as a part of a larger treatment regimen. Our findings on the use of radiation therapy align with a retrospective study of 18 terminal-stage MPS patients in which radiation therapy was found to provide long-term pain relief [12].

Three case reports utilized surgical intervention to treat MPS with mixed effectiveness in management [13,23]. One case showed no efficacy after computed tomography-guided cordotomy, while another utilized a subtotal vertebrectomy and resection of the left psoas muscle resulting in a disease-free status for three years [13,23]. These findings suggest that surgical therapy can alleviate MPS complications but should be used along with a multidisciplinary approach.

Limitations of Study

This scoping review has some limitations regarding the type of study used, sample size, selection of case reports with MPS, and data provided by the case reports. The inclusion criteria allowed for 20 case reports to be the only source of data extraction, which limited the information base of this study. In the selection process of the case reports and case series, several were selected as MPS patients by their symptoms, but they did not have a formally stated diagnosis of MPS. The limited sample size and source material of this study prevents any definitive claims, suggestions, or generalizations for MPS treatment from this article. Case reports within this review were limited by the lack of stated side effects, which restricted our conclusions to the effects of medications. Further, some case reports lacked information on specific opioid regimens or survival times of the patients following MPS diagnosis. Intractable MPS pain treatment is not limited to one treatment modality. Based on the literature reviewed, MPS pain management is a polypharmacy regimen involving variable doses, frequency of medication use, and consideration for various comorbidities; therefore, it is difficult to ascertain which specific treatments were effective in relieving pain. 

Implications for Future Practice

MPS is characterized as a condition resistant to many different pain management strategies that would otherwise be effective in other cancer-related conditions. This scoping review has shown anecdotal evidence that treatment modalities such as methadone, botulinum toxin, epidural administration of opioids, and radiation therapy are effective in managing the devastating symptoms of MPS. To build upon this anecdotal evidence, there is a need for more research on the effectiveness of each modality to create a unified, evidence-based pain management protocol for MPS. 

## Conclusions

The objective of this review was to identify case reports of MPS and map clinical criteria for diagnosis, treatment modalities, and the relative efficacy of each management strategy used. This review found that a diagnosis of MPS can be made amongst advanced cancer patients with clinical evidence of proximal lumbosacral plexopathy, fixed flexion of hip or associated limitation of movement and gait disorder, and imaging that demonstrates ipsilateral iliopsoas invasion. This review confirmed the difficulty in managing MPS with cases utilizing two to six different combinations of drug classes to manage symptoms. Many cases reported successful management of MPS symptoms through more aggressive strategies such as increased opioid dosage, epidural administration of opioids, nerve blocks, cordotomy, botulinum toxin injection, and radiation therapy. This scoping review suggests that a more standardized, evidence-based management protocol is required for MPS. Randomized controlled studies with greater sample sizes are suggested to establish an association between certain treatment modalities and a reduction in MPS symptoms.
